# The Role of TRESK in Discrete Sensory Neuron Populations and Somatosensory Processing

**DOI:** 10.3389/fnmol.2019.00170

**Published:** 2019-07-17

**Authors:** Greg A. Weir, Philippa Pettingill, Yukyee Wu, Galbha Duggal, Andrei-Sorin Ilie, Colin J. Akerman, M. Zameel Cader

**Affiliations:** ^1^Nuffield Department of Clinical Neurosciences, University of Oxford, Oxford, United Kingdom; ^2^Department of Pharmacology, University of Oxford, Oxford, United Kingdom

**Keywords:** TRESK, sensory neuron, pain, touch, somatosensory

## Abstract

Two-pore domain K^+^ (K_2P_) channels generate K^+^ leak current, which serves a vital role in controlling and modulating neuronal excitability. This diverse family of K^+^ channels exhibit distinct expression and function across neuronal tissues. TWIK-related spinal cord K^+^ channel (TRESK) is a K_2P_ channel with a particularly enriched role in sensory neurons and *in vivo* pain pathways. Here, we explored the role of TRESK across molecularly distinct sensory neuron populations and assessed its contribution to different sensory modalities. We found TRESK mRNA only in select populations of C- and A-δ nociceptors, in addition to low threshold D-hair afferents. Neurons from mice in which TRESK has been ablated demonstrated marked hyperexcitability, which was amplified under inflammatory challenge. Detailed behavioral phenotyping of TRESK knockout mice revealed specific deficits in somatosensory processing of noxious and non-noxious stimuli. These results demonstrate novel roles of TRESK in somatosensory processing and offer important information to those wishing to target the channel for therapeutic means.

## Introduction

Two-pore domain K^+^ (K_2P_) channels are the molecular mediators of K^+^ leak, which is essential for proper neuronal function. The conductance associated with K_2P_ activity stabilizes the resting membrane potential and attenuates depolarizing stimuli. As such, K_2P_ channels are becoming increasingly recognized as critical gatekeepers of neuronal excitability; important in controlling the initiation and form of the action potential (Enyedi and Czirják, 2010). Once considered passive ion conductors, K_2P_ channels are now known to be highly dynamic and regulated by a variety of factors, leading to an appreciation of their involvement in a range of physiological and pathological processes of the nervous system (Honoré, 2007; Plant, 2012; Steinberg et al., 2015).

TWIK-related spinal cord K^+^ channel (TRESK) is a K_2P_ channel with a prominent role in pain pathways. While expression data is somewhat conflicted, it is consistently reported that TRESK is highly expressed in sensory ganglia and that loss of channel activity results in enhanced sensory neuron excitability and increased activation of *in vivo* pain pathways (Dobler et al., 2007; Yoo et al., 2009; Tulleuda et al., 2011). TRESK expression is down-regulated following traumatic nerve injury and conversely, viral overexpression can ameliorate injury-induced pain hypersensitivity (Tulleuda et al., 2011; Zhou et al., 2013). Pharmacological inhibition of channel activity induces spontaneous pain behaviors in rodents (Tulleuda et al., 2011). There is also genetic evidence for a role of TRESK in human pain. A dominant negative frameshift mutation in *KCNK18*, the gene that encodes TRESK, was found to segregate in a pedigree of patients with typical migraine with aura (MA; Lafrenière et al., 2010). Activation of trigeminal nociceptors innervating the dural meninges likely underlies the headache phase of migraine (Weir and Cader, 2011), offering a plausible locus of action of the mutation due to the enrichment of TRESK in these afferents (Lafrenière et al., 2010). Such data is leading to increased efforts to target channel activity as an analgesic strategy (Wright et al., 2013; Bruner et al., 2014; Sehgal et al., 2014; Mathie and Veale, 2015).

A previous study characterizing functional TRESK knockout mice reported that channel activity accounts for 20% of the background K^+^ leak and limits the excitability of dorsal root ganglion (DRG) neurons (Dobler et al., 2007). A second TRESK knockout mouse has been shown to have a 20% increase in sensitivity to noxious thermal stimuli, as well as an enhanced susceptibility to volatile anesthetics (Chae et al., 2010). The relatively small effect sizes in these studies contrast with the fact that TRESK is the most abundant K_2P_ channel in sensory neurons (Dobler et al., 2007) and the aforementioned preclinical and genetic data implicating a substantive role in pain signaling. Sensory neurons are highly heterogeneous in their transcriptomes and perform distinct roles in sensory detection and processing (Cavanaugh et al., 2009; Usoskin et al., 2015). We do not yet have a clear understanding of which sensory neuron populations express TRESK. Furthermore, previous studies of mice with genetic ablation of TRESK have not evaluated function across the different neuronal populations, and have restricted behavioral analysis to noxious thermal sensitivity, but not other sensory modalities. In particular, no efforts have been made to assess TRESK KO mice for phenotypes that mirror the sensory dysfunctions common to migraineurs.

Given the clinical potential of therapies targeting TRESK, there is a need to better characterize the channels presence and role in different classes of sensory neurons. Here, we have followed up on detailed expression analysis of the sensory ganglia to define the consequence of TRESK ablation to select sensory neuron populations and the consequence of specific sensorimotor and nociceptive functions. We defined that TRESK is selectively expressed in sub-populations of nociceptors and low threshold D-hair afferents. Recordings from trigeminal ganglion (TG) neurons from TRESK knockout (TRESK [KO]) mice indicated enhanced neuronal excitability and an increased propensity to sensitize under inflammatory challenge. Finally, using behavioral studies, we found that TRESK [KO] mice display modality-specific deficits in tests of sensory function. Collectively, these results demonstrate a previously unappreciated specificity to the role of TRESK in somatosensory processing.

## Materials and Methods

### Animals

Mice were housed at the University of Oxford in holding rooms on a 12/12-h light/dark cycle (lights on at 07:00 h and off at 19:00 h) in single-sex groups of 2–6 with *ad libitum* food and water. Behavioral experiments were performed on mixed-sex naïve adult mice (14- to 20-week-old). This study was carried out in accordance with the principles of the United Kingdom Home Office Animals in Scientific Procedures Act (1986). The protocol was approved by the clinical medicine animal care and ethical review body, University of Oxford. TRESK KO and WT mice used in comparison studies were littermates. TRESK (GenBank accession number NM332396, Ensembl identification number ENSMUSG40901) knockout mice were obtained from the KOMP repository^1^. The VelociGene targeting system (Valenzuela et al., 2003) ablates *KCNK18* by replacing the majority of the coding region with a LacZ-neomycin cassette. Knockout mice were initially crossed with wild-type C57Bl/6J (Harlan, United Kingdom). Offspring from F5 heterozygous littermates were used in this study.

### RNA Isolation and Quantitative Real Time PCR

Mice were sacrificed using a CO_2_ chamber and TG was rapidly dissected and processed for RNA by RNeasy Mini Kit (QIAGEN) following the manufacturer’s instructions. RNA was reverse transcribed to cDNA by MutiScribe reverse transcriptase (Life Technologies, Carlsbad, CA, USA) following manufacturer’s instructions. Five microliter of template cDNA (20 ng/μl) was used for each 20 μl PCR reaction. Reaction utilized SYBER Green chemistry (Applied Biosystems, Foster City, CA, USA) and a StepOne Plus real-time PCR (RT-PCR) machine (Applied Biosystems, Foster City, CA, USA). Primers used in this study were: KCNK18 Fwd: 5′ CTCTCTTCTCCGCTGTCGAG 3′, KCNK18 Rev: 5′ AAGAGAGCGCTCAGGAAGG 3′, RPL19 Fwd: 5′ AGCCTGTGACTGTCCATTCC 3′, RPL19 Rev: 5′ GGCAGTACCCTTCCTCTTCC 3′. Relative expression of target genes was calculated using the comparative Ct (ΔΔCt) method.

### *In situ* Hybridization (ISH) and Immunohistochemistry

For tissue collection, animals were deeply anesthetized with pentobarbital and perfused with ice-cold 0.9% NaCl solution through the vascular system. TG were collected, post-fixed for 1–2 h in 4% paraformaldehyde (PFA) and stored in 30% sucrose for a minimum of 48 h before being embedded and sectioned by a cryostat (15 μm thick sections). *In situ* hybridization (ISH) was performed using the RNAScope 2.5 RED chromogenic assay kit, following the manufacturer’s instructions (Advanced Cell Diagnostics). Briefly, sectioned TG tissue was re-hydrated in PBS at RT followed by treatment with hydrogen peroxide at RT and a subsequent protease treatment in a hybridization oven at 40°C. Slides were then incubated with *KCNK18* mRNA probes (Advanced Cell Diagnostics) at 40°C for 2 h. Slides were then subjected to six rounds of amplification before the signal was developed by reaction with fast red. Samples were incubated at room temperature for 1 h in blocking solution (PBS + 0.3% Triton^TM^ X-100 + 10% normal goat serum), before overnight incubation with primary antibody at 4°C. Antibodies used in this study were as follows: NeuN (1:500, rabbit, ab177487, Abcam), NF200 (1:1,000, chicken, ab4680, Abcam), CGRP (1:1,000, rabbit, T-4032.0050, Peninsula Laboratories, San Carlos, CA, USA) IB4 (1:50, streptavidin conjugated, L2140, Sigma-Aldrich), parvalbumin (1:200, guinea pig, AF1000, Frontier Institute), TrkB (1:200, goat, R & D Systems, AF1494), TH (1:100, sheep, Millipore, AB1542), calbindin (1:200, rabbit, Swant, CB38) and TrkC (1:200, goat, R & D Systems, AF1404). Samples were then incubated with a corresponding fluorophore-conjugated secondary antibodies (1:1,000, Alexa Fluor^®^ 488), diluted in blocking solution for 2 h at RT. Samples were mounted and viewed by a Zeiss LSM-700 confocal microscope and ZEN software. Image analysis was performed using ImageJ (NIH). For image quantification, data were averaged from at least three sections per tissue and sampled from at least three animals per group. Only neurons with a clear nucleus were included in the analysis of TG cell size distribution. The intensity of red signal for each neuronal soma, manually defined as being positive for the sub-population marker, was measured. On each sample, background red signal was calculated by averaging the signal from three randomly selected regions that did not include neuronal somas. A neuron was defined as positive for TRESK expression if red signal intensity was >3 fold higher than mean background value.

### TG Dissociation

Mouse TG neuron cultures were prepared as outlined (Weir et al., 2017). Adult mice of either gender (4–12 weeks) were sacrificed and TG rapidly excised into ice-cold HBSS without Ca^2+^/Mg^2+^. Ganglia were diced into small pieces and incubated in Papain (20 U/ml—Worthington Biochemicals) solution for 20 min at 37°C. Further enzymatic dissociation involved a second 20 min incubation in Dispase type II/Collagenase solution (Worthington Biochemicals). Cells were washed to remove the residual enzyme and mechanically dissociated through a fire-polished glass pipette. Cells were plated onto laminin/poly-D lysine treated coverslips in Neurobasal media supplemented with 2% (v/v) B27, 1% (v/v) P/S (ThermoFisher), 50 ng/ml mouse NGF (Preprotech) and 10 ng/ml GDNF (Preprotech). Cells were used for recordings 24–72 h after plating.

### CGRP Release ELISA

Primary TG neurons from WT (*n* = 3) and TRESK-KO mice (*n* = 3) were plated in technical duplicates in a 24-well plate format as above. Neurons were stimulated for 30 min with 50 mM KCl and the media was collected, centrifuged at 200 g for 5 min and supernatants were frozen at −80°C. Basal media was collected prior to KCl-stimulation from each well to serve as a baseline. CGRP ELISA was conducted using Rat CGRP Enzyme Immunoassay Kit as per manufacturer’s instruction (Bioquote). Plates were read on a Wallac Victor 1420 Multilabel counter at 405 nm for 0.1 s and data was analyzed on GraphPad Prism 7 software.

### Electrophysiology

Patch-clamp experiments were performed in whole-cell configuration at room temperature using an Axopatch 200D amplifier controlled by pClamp 10 software (Molecular Devices). Patch pipettes had resistances between 2 MΩ and 4 MΩ. Extracellular solution contained (in mM): 140 NaCl, 4.7 KCl, 2.5 CaCl_2_, 1.2 MgCl_2_, 10 HEPES and 10 glucose; pH was adjusted to 7.4 with NaOH. The pipette was filled with a solution containing (in mM): 130 KCl, 1 MgCl_2_, 5 MgATP, 10 HEPES and 0.5 EGTA; pH was adjusted to 7.3 with KOH. The osmolarity of all solutions was maintained at 315 mOsm/L for extracellular solution and 305 mOsm/L for intracellular solutions. Cell capacitance and series resistance were constantly monitored throughout the recording. Data were analyzed with Clampfit 10 (Molecular Devices). To assess IB4 binding, cells were incubated with 10 μg/ml Fluorescein labeled isolectin B4 (Vector Laboratories) for 30 min prior to recording. Such labeling has previously been shown to not alter electrophysiological properties of small diameter DRG neurons (Stucky and Lewin, 1999).

#### Current Clamp

Two minutes after establishing whole-cell access baseline biophysical properties such as cell capacitance, resting membrane resistance and input resistance were measured. R_input_ was calculated by measuring the membrane potential change induced by hyperpolarizing current injection from 20 pA to 100 pA. R_input_ and all evoked firing protocols were performed from a holding potential of −60 mV. For analysis of rheobase, short (50 ms) injections of depolarizing current were increased incrementally (Δ10 pA) until an action potential was fired. To assess repetitive firing, response to prolonged (500 ms) depolarizing current injection (25–350 pA) were monitored. Spontaneous firing was recorded in bridge mode while zero current was inputted. For sensitization experiments inflammatory soup (IS) contained; 50 nM bradykinin, 500 nM PGE_2_, 2 μM ATP, 500 nM noradrenaline and 1 μM histamine (Sigma Aldrich).

### Calcium Imaging

Cell media was supplemented with 2 μM Fura-2 AM and 80 μM pluronic acid (ThermoFisher Scientific) for 1 h at 37°C, before being transferred to the recording solution, which contained (in mM): 145 NaCl, 5 KCl, 10 HEPES, 10 glucose, 2 CaCl_2_ and 1 MgCl_2_, pH was adjusted to 7.4. A 10× objective, dichroic LP 409 mirror and BP 510/90 emitter filter were used for all imaging. Pairs of images using excitation filters BP 340/30 and BP 387/15, respectively, were captured every 2 s. Ratiometric 340/380 calculation was performed with a background subtraction. IB4^+^ TG were labeled as above and identified using a BP 480/30 excitation filter, dichroic LP 505 mirror and BP 535/40 emission filter. Recording solution was perfused with time-locked addition of IS/50 mM KCl by a gravity-driven application system with remotely controlled pinch valves. Cells exhibiting a Ca^2+^ transient >0.2 ΔF/F directly following treatment were defined as having responded.

### Behavioral Protocols

All behavioral studies were performed by an experimenter blinded to genotype and at consistent time of day. Prior to testing, mice were acclimatized to the testing equipment and values were obtained by averaging data from two to three sessions for each test.

#### Locomotor Function

Balance and coordination were examined* via* the locomotor test. Mice were placed on the rotarod (#7650, Ugo Basile) from 2 to 40 rpm over a period of 300 s and the speed at which the mouse fell was recorded.

#### Non-anxiogenic Open Field

The open field consists of a dark arena (dimensions height × width, 50 × 30 cm) divided into 10 cm squares. Mice were placed into the arena for 3 min and the number of squares entered, the number of rears and the latency to rear recorded.

#### Von Frey

Static mechanical thresholds in freely-moving awake animals were examined* via* von Frey hair application (0.008–1 g; Touch Test; Stoelting) to the plantar surface of the hindpaw using the “up–down” method (Chaplan et al., 1994). Before testing, mice were randomly assigned into individual Plexiglas cubicles (8 × 5 × 10 cm) for 1 h on an elevated wire mesh floor to enable access to the paw surface. Calibrated von Frey hairs were applied to the left and right hind paws, starting with the 0.6 g filament, until the fiber bowed. A positive withdrawal response is followed by a lower force hair and vice versa for a negative withdrawal response until a change in behavior occurs. Using this up–down sequence, four subsequent hairs were assessed and the 50% paw-withdrawal threshold (PWT) was calculated as described by Dixon (1980). Data presented are from both left and right hind paws averaged together as no laterality effects were observed.

#### Hargreaves

Thermal thresholds in freely-moving awake mice were assessed with the Hargreaves method using the plantar test (37370, UgoBasile). Before testing, mice were assigned to testing cubicles at random and habituated to the apparatus for 1 h in individual cubicles (8 × 5 × 10 cm) placed on a glass plate. An infrared light source was applied to the plantar surface of hind-paws through the glass plate. Withdrawal reflexes were recorded from both left and right-paws, on three occasions, leaving at least 2 min between stimuli.

#### Hot Plate

Noxious-thermal thresholds of the hind-paws were examined* via* the hot plate test using an incremental hot/cold plate (35150, UgoBasile) set at a temperature of 53°C ± 0.2°C. Mice were placed in the center of the hot plate in a 10 cm diameter testing box; licking, jumping or stamping reflex responses were recorded. A maximum latency of 20 s was permitted to prevent tissue damage.

#### Photophobia

Mice were individually tested in a light/dark box (Crawley, 1981), which consisted of a dark enclosed compartment with a removable lid (15 × 20 × 20 cm, 0 lux) and a brightly-lit white-compartment (30 × 20 × 20 cm, 900 lux). A small opening (3 × 3 cm) connects the two compartments allowing animals to move freely. Prior to testing mice were acclimatized for at least 30 min in their home cages to the testing room. Each mouse was gently placed into the center of the light compartment, facing away from the opening. Behaviors were scored for 5 min, including the latency to cross into the dark compartment (defined by all four paws in that area), time spent in each compartment and the number of transitions through compartments were scored. The light/dark box was thoroughly cleaned with 95% EtOH between animals.

#### Cotton Swab

Mice were housed in plexiglass cubicles on elevated wire-mesh floor as per the Von frey test. A “puffed out” cotton swab was stroked over the plantar surface of the hindpaw at a consistent speed (~0.5 s) five times. The number of withdrawals in response to five strokes per paw were recorded and averaged between the two paws.

#### Back Tape Test

A 3 cm piece of sticky tape (Starlab autoclave tape, 25 mm × 50 mm) was placed firmly to the back of each mouse. The number of bouts (scratching and licking) directed at the tape was recorded over a 5 min period while the mouse was housed in a plexiglass cubile.

### Statistics

The data are expressed as mean ± standard error of the mean (SEM). In cases where data did not fit the conditions of parametric testing (homogeneity of variance and normality of sampling), nonparametric tests were used. Statistical analysis was performed using GraphPad PRISM and the significance level was set at *p* < 0.05.

## Results

### TRESK Is Selectively Expressed in Discrete Populations Within the Trigeminal Ganglion

**Figure 1:**
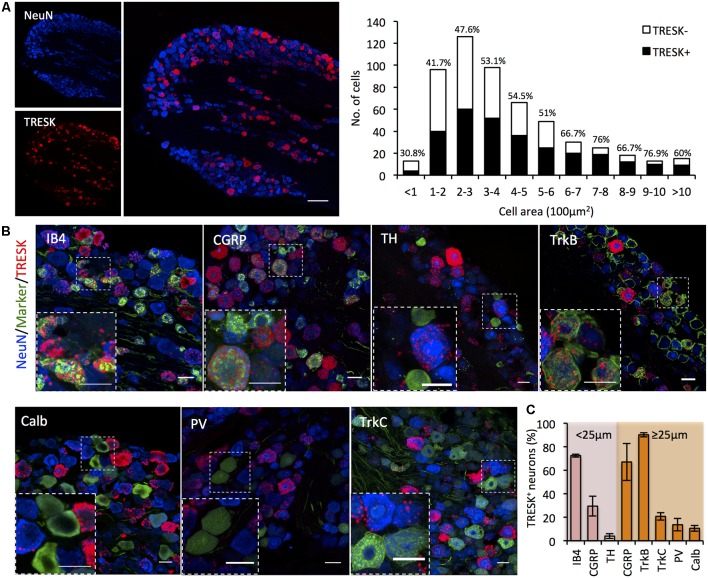
*KCNK18* mRNA in mouse trigeminal ganglion (TG). *In situ* hybridization for *KCNK18* mRNA combined with immunocytochemistry for molecular markers of canonical sensory neuron populations. **(A)** *Left*, Overview image of *KCNK18* mRNA (red) in TG stained for NeuN (blue). Scale bar represents 100 μm. *Right*, size-frequency distribution of *KCNK18* positive neurons. Expression was defined as any neuron with signal >3-fold mean background. Data pooled from three animals and from at least three sections per animals (total of 521 neurons). **(B)** Representative images of *KCNK18* mRNA (red) with population markers (green) and all neurons marked by NeuN (blue). Insets show magnified view of selected region. All scale bars represent 25 μm. **(C)** Quantification of *KCNK18* mRNA expression in different populations. Neurons are categorized as small diameter (≤25 μm) or medium/large (>25 μm). Data represents mean ± standard error of the mean (SEM) from* n* = 3 animals and at least three section per marker and animal. PV, Parvalbumin, Calb, calbindin; TH, tyrosine hydroxylase; and CGRP, calcitonin gene related peptide.

In human and rodent tissue, TRESK is highly enriched in sensory ganglia of the DRG and TG (Dobler et al., 2007; Lafrenière et al., 2010). Previous studies report that >90% of sensory neurons in the DRG express TRESK (Dobler et al., 2007; Yoo et al., 2009), however single-cell RNA sequencing data challenges the assertion that expression is as widespread (Usoskin et al., 2015). We used RNA ISH to define TRESK expression in the TG and found expression to be highly heterogeneous across neurons. TRESK mRNA was present in 51.14 ± 4.56% of all neurons. Size-frequency analysis revealed that TRESK was present in neurons of all soma diameters (Figure 1A). We combined ISH with immunohistochemistry to examine expression across molecularly defined sub-populations. Within small diameter neurons (≤25 μm) that likely represent unmyelinated C-fibers, TRESK was present in 72.43 ± 1.25% of neurons that bound the lectin IB_4_ (non-peptidergic nociceptors), but only 29.43 ± 8.50% of calcitonin gene related peptide (CGRP) positive neurons (peptidergic nociceptors; Nagy and Hunt, 1982; Silverman and Kruger, 1990). Expression was virtually absent in tyrosine hydroxylase (TH) positive, C-low threshold mechanoreceptors (3.98 ± 2.03%; Li et al., 2011). Within medium/large diameter neurons (≥25 μm), we observed expression in a large proportion of CGRP^+^ neurons (66.98 ± 15.70%) and neurons highly expressing TRKB^+^ (90.15 ± 1.74%), representing a subset of Aδ-nociceptors and D-hairs respectively (Lawson et al., 2002; Li et al., 2011). In contrast, we saw minimal expression in Calbindin^+^ (10.62 ± 2.51%) or TRKC^+^ (20.74 ± 3.27%) neurons, representing populations of Aβ-low threshold mechanoreceptors (Usoskin et al., 2015). Few proprioceptors, classified as large-diameter neurons expressing parvalbumin, expressed TRESK (13.62 ± 5.25%; Figures 1B,C). In sum, these results demonstrate the heterogeneity of TRESK expression across different TG populations and highlight the need to study protein function in specific sub-populations.

### TRESK Selectively Modulates Non-peptidergic Small Diameter Nociceptor Excitability

**Figure 2:**
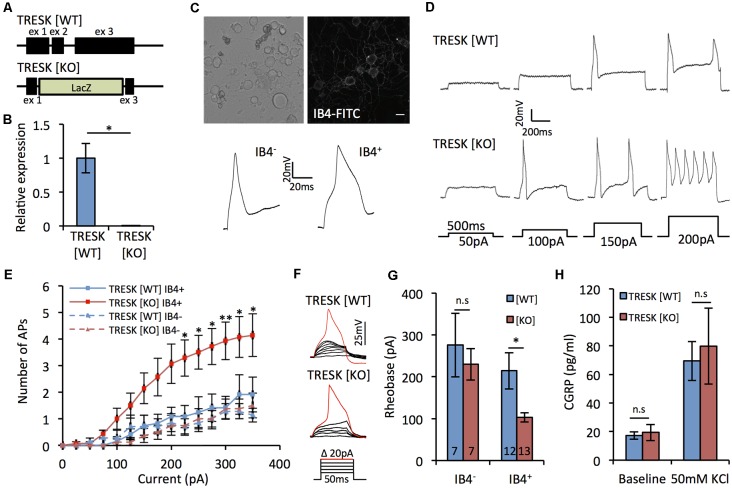
TRESK selectively modulates non-peptidergic C-fiber excitability. **(A)** Targeted ablation of *KCNK18* gene. Allele describes neomycin/lacZ cassette, which replaces 3′ of exon 1, all of exon 2 and 5′ exon 3. **(B)** Real time PCR (RT-PCR) for TRESK expression in dorsal root ganglion (DRG) mRNA. Data derived from* n* = 3 animals. *p < 0.05, student’s unpaired *t*-test. **(C)** Neurons dissociated 24–48 h previously from TRESK [WT] and TRESK [KO] TG were used for patch clamp recordings. Example image of neurons live stained for IB4-FITC binding and typical action potential waveforms generated from IB4^+^ and IB4^−^ neurons. Note the prolonged duration of the action potential of IB4^+^ neurons. **(D)** Example evoked firing profiles of IB4^+^ TG neurons of both genotypes in response to prolonged (500 ms) depolarizing steps. **(E)** Quantification of TG neuron firing in response to prolonged (500 ms) suprathreshold current stimulation. **p < 0.01, *p < 0.05, RM two-way analysis of variance (ANOVA) with Sidak multiple testing comparing TRESK [KO] IB4^+^ and TRESK [WT] IB4^+^ groups. Data represents mean ± SEM from 12 (TRESK [WT] IB4^+^), 7 (TRESK [WT] IB4^−^), 13 (TRESK [KO] IB4^+^) and 7 (TRESK [KO] IB4^−^) neurons. Recordings were pooled from TG obtained from two mice (per genotype) cultured independently on two separate days. **(F)** Example evoked firing profiles of IB4^+^ TG neurons of both genotypes in response to short (50 ms) depolarizing steps. **(G)** Rheobase of TG neurons, defined as the minimum current of 50 ms duration required to generate an action potential. *p < 0.05, Kruskal-Wallis followed by Dunn’s multiple comparisons test. Same sample sizes as **(E)**, **(H)** CGRP released into culture media at baseline and during activation with 50 mM KCl containing media. Data derived from *n* = 3 animals, each with technical triplicates. n.s, *p* > 0.05. One-way ANOVA comparing genotypes for each condition.

When sensory neurons from TRESK knockout mice have been assessed non-selectively, the channel has been shown to act as a molecular brake on excitability (Dobler et al., 2007). Given our expression data, we sought to explore whether global loss of TRESK activity had population-specific effects on excitability. The majority of the coding region of *KCNK18*, the gene that encodes TRESK, was targeted for deletion (Figure 2A), resulting in TRESK KO animals with no TRESK transcripts (Figure 2B). As a population in which TRESK is abundantly expressed, we performed recordings of small-diameter TG neurons live-stained for IB_4_ binding, which demarks non-peptidergic nociceptors. Small neurons that do not bind IB4 will comprise peptidergic nociceptors and C-low threshold mechanoreceptors, two populations in which our *in situ* data would suggest that TRESK expression is minimal. IB_4_^+^ neurons exhibited broadened action potentials (Figure 2C), consistent with high levels of Na_V_1.9 (Fjell et al., 1999; Stucky and Lewin, 1999). Excitability, both in terms of the number of action potentials fired in response to suprathreshold stimuli and the current threshold for initial action potential generation, was enhanced in TRESK [KO] IB_4_^+^ neurons, but not IB_4_^−^ TRESK [KO] neurons, in comparison to TRESK [WT] (Figures 2D–G). We observed no difference between the genotypes for resting membrane potential ([WT] −47.2 ± 2.3 mV vs. [KO] −48.1 ± 1.8 mV; *p* > 0.05 Mann-Whitney *U* test) or input resistance ([WT] 245.8 ± 28.5 MΩ vs. [KO] 275.1 ± 24.8 MΩ; *p* > 0.05 Student’s *t*-test). Further supporting a limited role in peptidergic neurons, there was no difference in release of CGRP from TRESK [KO] cultures during basal or stimulated (50 mM KCl) conditions (Figure 2H). These results align with the expression data and suggest that within small diameter nociceptors, TRESK selectively operates in non-peptidergic neurons to provide substantial inhibitory tone.

### Lack of TRESK Exaggerates Inflammatory Sensitization

**Figure 3:**
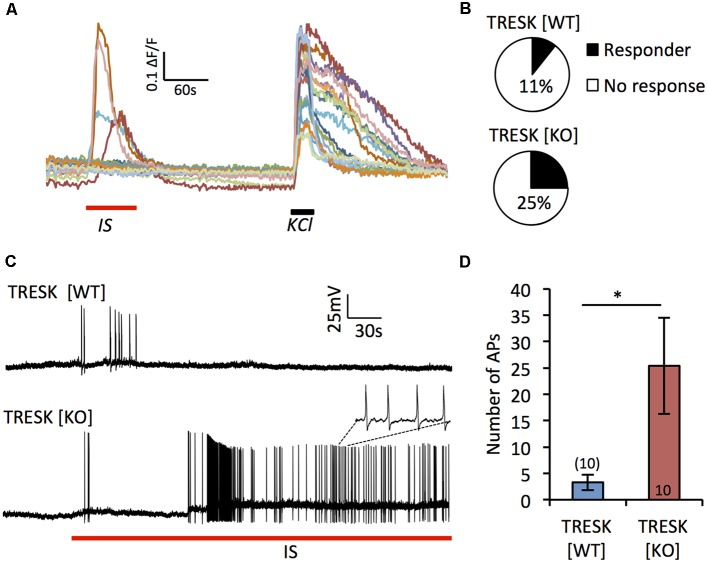
Loss of TRESK increases activation in response to inflammatory stimulation. **(A)** Representative traces of Ca^2+^ signals in TRESK [KO] TG neurons exposed to inflammatory soup (IS; 1 min) followed by a 5 min wash out and then 50 mM KCl to depolarize all neurons (30 s). **(B)** Proportion of IB4^+^ TG neurons from each genotype responding to IS with a Ca^2+^ peak >0.2 ΔF/F (TRESK [WT]* n* = 45, TRESK [KO] *n* = 97 cells). **(C)** Patch clamp recordings of TG neuron firing in response to acute (5 min) exposure to IS. **(D)** Quantification of action potential discharge during the 5 min of inflammatory challenge. Data represents mean ± SEM from 10 recordings per group. Recordings were pooled from TG obtained from three mice (per genotype), cultured independently on three separate days. *p < 0.05, student’s unpaired *t*-test.

TRESK has been demonstrated to be both positively and negatively regulated by inflammatory factors (Callejo et al., 2013; Kollert et al., 2015). To address the role TRESK plays in nociceptor sensitization, we acutely applied IS (bradykinin, PGE_2_, noradrenaline, ATP and histamine) to TG neurons (Figure 3A). In response to IS, a greater proportion of TRESK [KO] IB4^+^ nociceptors demonstrated a Ca^2+^ influx compared to TRESK [WT] IB4^+^ neurons (Figure 3B; TRESK [WT] 4/45 cells vs. TRESK [KO] 30/97 cells, *p* = 0.0053 Fischer’s exact *t*-test). Paired-patch clamp recordings confirmed the greater propensity for TRESK [KO] neurons to activate when challenged with IS. In response to acute IS treatment, IB4^*+*^ TRESK [KO] neurons fired >7-fold more action potentials than TRESK [WT] neurons (Figures 3C,D; TRESK [WT] 3.3 ± 0.1.48 vs. TRESK [KO] 25.4 ± 9.16 action potentials, *p* = 0.039 student’s unpaired *t-test*). These results indicate that TRESK activity reduces *in vitro* inflammatory sensitization of nociceptors.

### Sensorimotor Testing of TRESK Knockout Mice

**Figure 4:**
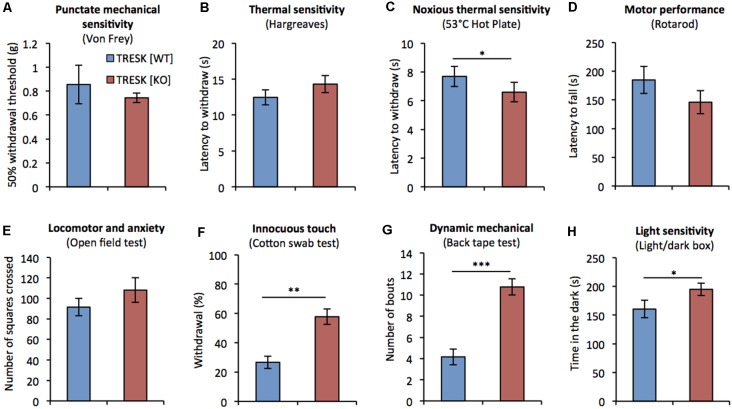
TRESK [KO] mice exhibit selective deficits in sensory behavior. Sensorimotor testing of adult TRESK [KO] mice (red bars) and TRESK [WT] littermate controls (blue bars). **(A)** Punctate mechanical sensitivity as assessed by withdrawal to Von Frey filaments of different force. Data represents mean ± SEM from 16 TRESK [WT] and 11 TRESK [KO] animals. **(B)** Latency to withdraw hindpaw to radiant thermal stimuli (Hargreaves test). Cut-off was 30 s. **(C)** 53°C hot plate test of noxious thermal sensitivity. Cut-off was 20 s. **(D)** Latency of mice to fall on a rotating beam (rotarod test). **(E)** Number of squares crossed by mice in the open field test of locomotion and anxiety. **(F)** Number of scratches/bites in response to 5 cm sticky tape applied to the back of mice over a 5 min period. Data represents mean ± SEM from six TRESK [WT] and nine TRESK [KO] animals. **(G)** Number of withdrawals to five gentle brushes of the hindpaw with a “fluffed” cotton bud. Data represents mean ± SEM from six TRESK [WT] and nine TRESK [KO] animals. **(H)** Time spent in the dark during 5 min of testing in the light/dark box test. Unless otherwise specified, data represent mean ± SEM from 15 (TRESK [WT]) and 14 (TRESK [KO]) animals and statistical comparisons between genotypes were performed by a Mann-Whitney-*U* test. ***p < 0.001, **p < 0.01, *p < 0.05.

Previous assessment of mice lacking TRESK has been restricted to testing thermal nociception and has not assessed mechanical, or non-nociceptive sensitivity (Chae et al., 2010). We examined the behavior of TRESK [KO] mice in response to a range of sensory challenges. TRESK [KO] mice had normal growth and showed no structural abnormalities; body weight was normal compared to TRESK [WT] littermates ([♂ WT] 25.81 ± 0.4 g vs. [♂ KO] 25.67 ± 0.6 g and [♀ WT] 18.85 ± 0.5 g vs. [♀ KO] 18.93 ± 0.8 g; *p* > 0.05 unpaired student’s *t*-test, *n* = 6, 8–10 week old littermate mice per group). TRESK [KO] mice exhibited normal punctate mechanical sensitivity as assessed by Von Frey filaments (Figure 4A). Plantar thermal sensitivity was normal when measured by Hargreaves (Figure 4B), but TRESK [KO] mice demonstrated a 14% decrease in time to respond on the nociceptive 53°C hot-plate test (Figure 4C, [WT] 7.7 ± 0.7 s vs. [KO] 6.6 ± 0.7 s; *p* = 0.039, Mann-Whitney *U* test). Genotype had no effect on motor performance or locomotor activity, as assayed by the Rotarod and Open field tests respectively (Figures 4D,E).

Given the enriched expression of TRESK in the D-hair population, which are low-threshold mechanoreceptors that activate to extremely light touch, we tested whether TRESK [KO] mice exhibited heightened sensitivity during a light touch behavioral assay (Garrison et al., 2012). TRESK [KO] mice were more likely to respond to light brushing of the hindpaw with a cotton swab (Figure 4F, [WT] 26.7 ± 4.1% responding vs. [KO] 57.8 ± 5.2%; *p* = 0.002 Mann-Whitney *U* test). In a test of dynamic mechanical sensitivity, TRESK [KO] mice exhibited an exaggerated response to adhesive tape placed on the back (Ranade et al., 2014; Figure 4G, [WT] 4.2 ± 0.7 bouts vs. [KO] 10.8 ± 0.8; *p* = 0.0004 Mann-Whitney *U* test). These results illustrate that TRESK expression regulates behavioral responses to extremely light static, and dynamic mechanical stimuli.

Migraine patients have higher sensitivity to light than non-migraneurs, even during migraine-free periods (Drummond, 1986). We sought to evaluate photophobia in TRESK [KO] mice under basal conditions. TRESK [KO] mice spent significantly more time compared to TRESK [WT] mice (Figure 4H, [WT] 160 ± 15.37 s vs. [KO] 194.8 ± 10.85 s; *p* = 0.0412 unpaired Student’s *t*-test) in the dark enclosed-compartment of the light/dark box, suggestive of heightened light sensitivity.

## Discussion

The enriched expression of TRESK in sensory ganglia and the role of TRESK in moderating neuronal excitability suggest an important function in nociceptive transmission. In support, preclinical injury models and human genetic studies suggest that loss of TRESK activity can contribute to pathological pain (Lafrenière et al., 2010; Tulleuda et al., 2011; Zhou et al., 2013). We have performed a detailed analysis of TRESK expression within sensory ganglia and define striking heterogeneity across sub-populations. In populations in which TRESK is enriched, genetic ablation leads to marked hyperexcitability, which is exacerbated by *in vitro* nociceptor sensitization following inflammatory challenge. At the behavioral level, mice lacking TRESK exhibit very specific sensory deficits, which correlate well with the known function of populations in which TRESK is enriched.

Reports of TRESK expression are conflicted, but consistent through all studies is high expression in sensory ganglia (Sano et al., 2003; Dobler et al., 2007; Yoo et al., 2009; Lafrenière et al., 2010). Sensory neurons are highly heterogeneous and distinct sub-populations likely perform specialized roles in somatosensation. Information on expression across sub-populations is therefore very important to understand gene function. Our data contradict previous assertions that TRESK is homogenously expressed in the sensory ganglia (Dobler et al., 2007; Yoo et al., 2009), and instead demonstrates that expression is restricted to select sub-populations. We believe that the contradictory results may be a result of refined ISH technologies used in this study, which allow for more sensitive and specific detection of mRNA expression and are being increasingly exploited in sensory biology (Wang et al., 2012; Dawes et al., 2018; Dembo et al., 2018). Our data is highly congruous with recent single-cell RNA-sequencing studies which describe TRESK to be enriched primarily in non-peptidergic nociceptors (NP1 and NP2), but also present in Aδ-nociceptors (PEP2) and D-hair afferents (NF1) and to a lesser extent in C-LTMRs (TH; which we did not observe; Usoskin et al., 2015; Zeisel et al., 2018). We cannot rule out the possibility that by setting a specific threshold of expression that we are finding false negatives. However, we observed no functional effect of genetic ablation in populations that we defined to lack TRESK expression, suggesting that even if very low levels of TRESK is present in these populations, it does not have a functional role.

TRESK has recently been demonstrated to heterodimerize with TREK1/2 channels in heterologous expression systems (Royal et al., 2019). In elegant studies, the same authors describe a mechanism by which the migraine-associated frameshift mutation (F139WfsX24) leads to production of a novel secondary protein product that selectively inhibits TREK1/2 channels and proposed that this effect of the mutation underlies migraine pathogenicity. Intriguingly, the study found that overexpression of a mutant protein product with strong dominant negative effects on wildtype TRESK channels had no effect on TG excitability. This is in contrast to previous studies and our own, which describe large changes in sensory neuron excitability upon loss of TRESK current (Dobler et al., 2007). Our results suggest that the negative findings described by the authors could in part be explained by recording from many neurons lacking endogeneous TRESK. Single-cell data suggests a degree of overlap of TRESK and TREK2 in sub-populations of non-peptidergic neurons (NP2) and Aδ-nociceptors (PEP2), however TREK1 expression shows little overlap (Zeisel et al., 2018). Future studies will be needed to confirm the profile of TREK1/2 co-expression in TRESK^+^ populations as this may help define primary afferent subtypes important in migraine pathogenesis.

Given the prominent role TRESK plays in dampening neuronal activity, loss of channel function could be expected to enhance hyperexcitability in pathological states. We show that TG neurons lacking TRESK exhibit increased action potential discharge in response to an inflammatory challenge commonly used in experimental models of migraine and pain. Inflammatory factors have been shown to both inhibit and activate TRESK (Callejo et al., 2013; Kollert et al., 2015), and as such it is not clear whether increased neuronal activation is due to a direct modulatory effect of IS on TRESK currents, or whether loss of the inhibitory drive of TRESK activity is simply additive to the excitatory drive of IS treatment. Irrespective of this, our data suggest that TRESK activity plays a role in nociceptor sensitization by inflammation.

TRESK [KO] mice were hypersensitive to noxious thermal stimuli, which is proposed to be encoded by peptidergic afferents (McCoy et al., 2013; Cowie et al., 2018). While we did not observe significant TRESK expression in small diameter peptidergic fibers, we did see high expression in medium/large sized peptidergic neurons. These neurons likely represent Aδ-nociceptors, some of which are capable of responding to noxious thermal stimuli (Koltzenburg et al., 1997). A second population of peptidergic Aδ-nociceptors has been shown recently to respond to high-threshold mechanical stimuli induced by pulling of a single hair (Ghitani et al., 2017). Dysfunction in this population could be linked to the exaggerated response to sticky tape applied to the hairy skin of the back that we observed in TRESK KO mice. Of our sensory behavioral assays, we observed the biggest deficiency in TRESK KO animals when innocuous touch was assessed. We used a cotton swab to assess response to light stroke, an assay which has been shown sensitive to detect dysfunction in light-touch cutaneous afferents (Garrison et al., 2012). D-hair afferents are the most sensitive mechanoreceptors of the skin (Lechner and Lewin, 2013), can be marked by high TrkB expression, and we found to express TRESK abundantly. Mice in which TrkB^+^ afferents have been ablated are less responsive to cotton swab, implicating this population in our TRESK KO phenotype. Aberrant D-hair afferent transmission has been associated recently with mechanical hypersensitivity following nerve injury (Peng et al., 2017; Dhandapani et al., 2018), which may help explain the link between loss of TRESK and enhanced mechanical hypersensitivity following traumatic nerve injury (Tulleuda et al., 2011; Zhou et al., 2013).

We cannot rule out the possibility that the method of genetic ablation (targeted insertion of a lacZ/neomycin cassette into *KCNK18* locus) disrupts the expression of genes other than *KCNK18* and contributes to the behavioral phenotype documented. However, the *in vitro* and *in vivo* phenotypes we observed are entirely consistent with predicted TRESK function, giving us confidence that they are mediated through the loss of the channel. In KO animals, TRESK is ablated globally rather than in a tissue-specific manner. Therefore, loss of TRESK function in structures other than primary afferents could be expected to influence behavioral phenotypes. TRESK expression has been noted in regions of rat brain and spinal cord (Yoo et al., 2009; Hwang et al., 2015). However, recent single-cell sequencing data profiling the mouse nervous system describes TRESK expression to be restricted to primary afferents and some neurons of the sympathetic nervous system (Zeisel et al., 2018) and to be absent from all neuronal subtypes in the dorsal horn of the spinal cord (Häring et al., 2018). We did not observe obvious signs of cognitive dysfunction in TRESK KO mice and animals performed normally in the Open Field Test. These data, in conjunction with the specific deficits in sensory behavior that we observed, strongly suggest that the phenotypes we describe following TRESK ablation are mediated by primary afferent dysfunction.

Taken together, our data show that contrary to prior assumptions, TRESK is selectively expressed in distinct sensory sub-populations and that it underlies very specific roles in somatosensory processing. TRESK is actively being pursued as a promising therapeutic target, given its important role in sensory neuron excitability. Its influence on non-nociceptive afferents has relevance for drug design. Under basal conditions these afferents code for innocuous light touch. However, in pathological conditions, their altered profile may mediate mechanical hypersensitivity- a prominent symptom of neuropathic pain and migraine. Thus, TRESK is an important mediator of nociceptive transmission and future studies investigating its role in pathological pain states may be fruitful.

## Data Availability

All datasets generated for this study are included in the manuscript.

## Ethics Statement

The maintenance and testing of these animals were performed in accordance with the United Kingdom Home Office Animals in Scientific Procedures Act (1986), at a licensed facility within the University of Oxford and following institutional review board approval.

## Author Contributions

GW, PP and MC: conceptualization. GW, PP, YW, GD, A-SI, CA and MC: methodology, writing—review and editing. GW, PP, YW and GD: investigation. GW: writing—original draft. GW, PP and YW: visualization. MC and CA: supervision and funding acquisition.

## Conflict of Interest Statement

MC has received honoraria and consultancy from Novartis, TEVA, Orion and Daichii Sankyo. The remaining authors declare that the research was conducted in the absence of any commercial or financial relationships that could be construed as a potential conflict of interest.

## Footnotes

^1^www.komp.org

## References

[B1] Bruner J. K. Zou B. Zhang H. Zhang Y. Schmidt K. Li M. (2014). Identification of novel small molecule modulators of K_2P_18.1 two-pore potassium channel. Eur. J. Pharmacol. 740, 603–610. 10.1016/j.ejphar.2014.06.021 24972239 PMC4222048

[B2] Callejo G. Giblin J. P. Gasull X. (2013). Modulation of TRESK background K^+^ channel by membrane stretch. PLoS One 8:e64471. 10.1371/journal.pone.0064471 23691227 PMC3655163

[B3] Cavanaugh D. J. Lee H. Lo L. Shields S. D. Zylka M. J. Basbaum A. I.. (2009). Distinct subsets of unmyelinated primary sensory fibers mediate behavioral responses to noxious thermal and mechanical stimuli. Proc. Natl. Acad. Sci. U S A 106, 9075–9080. 10.1073/pnas.0901507106 19451647 PMC2683885

[B4] Chae Y. J. Zhang J. Au P. Sabbadini M. Xie G. X. Yost C. S. (2010). Discrete change in volatile anesthetic sensitivity in mice with inactivated tandem pore potassium ion channel TRESK. Anesthesiology 113, 1326–1337. 10.1097/aln.0b013e3181f90ca5 21042202 PMC3010361

[B100] Chaplan S. R. Bach F. W. Pogrel J. W. Chung J. M. Yaksh T. L. (1994). Quantitative assessment of tactile allodynia in the rat paw. J. Neurosci. Methods 53, 55–63. 7990513 10.1016/0165-0270(94)90144-9

[B102] Crawley J. N. (1981). Neuropharmacologic specificity of a simple animal model for the behavioral actions of benzodiazepines. Pharmacol. Biochem. Behav. 15, 695–699. 6118883 10.1016/0091-3057(81)90007-1

[B5] Cowie A. M. Moehring F. O’Hara C. Stucky C. L. (2018). Optogenetic inhibition of CGRPα sensory neurons reveals their distinct roles in neuropathic and incisional pain. J. Neurosci. 38, 5807–5825. 10.1523/JNEUROSCI.3565-17.2018 29925650 PMC6010565

[B6] Dawes J. M. Weir G. A. Middleton S. J. Patel R. Chisholm K. I. Pettingill P.. (2018). Immune or genetic-mediated disruption of CASPR2 causes pain hypersensitivity due to enhanced primary afferent excitability. Neuron 97, 806.e10–822.e10. 10.1016/j.neuron.2018.01.033 29429934 PMC6011627

[B7] Dembo T. Braz J. M. Hamel K. A. Kuhn J. A. Basbaum A. I. (2018). Primary afferent-derived BDNF contributes minimally to the processing of pain and itch. eNeuro 5:ENEURO.0402–18.2018. 10.1523/eneuro.0402-18.2018 30627644 PMC6325548

[B8] Dhandapani R. Arokiaraj C. M. Taberner F. J. Pacifico P. Raja S. Nocchi L.. (2018). Control of mechanical pain hypersensitivity in mice through ligand-targeted photoablation of TrkB-positive sensory neurons. Nat. Commun. 9:1640. 10.1038/s41467-018-04049-3 29691410 PMC5915601

[B9] Dobler T. Springauf A. Tovornik S. Weber M. Schmitt A. Sedlmeier R.. (2007). TRESK two-pore-domain K^+^ channels constitute a significant component of background potassium currents in murine dorsal root ganglion neurones. J. Physiol. 585, 867–879. 10.1113/jphysiol.2007.145649 17962323 PMC2375503

[B10] Drummond P. D. (1986). A quantitative assessment of photophobia in migraine and tension headache. Headache 26, 465–469. 10.1111/j.1526-4610.1986.hed2609465.x 3781834

[B101] Dixon W. J. (1980). Efficient analysis of experimental observations. Annu. Rev. Pharmacol. Toxicol 20, 441–462. 10.1146/annurev.pa.20.040180.002301 7387124

[B11] Enyedi P. Czirják G. (2010). Molecular background of leak K^+^ currents: two-pore domain potassium channels. Physiol. Rev. 90, 559–605. 10.1152/physrev.00029.2009 20393194

[B12] Fjell J. Cummins T. R. Dib-Hajj S. D. Fried K. Black J. A. Waxman S. G. (1999). Differential role of GDNF and NGF in the maintenance of two TTX- resistant sodium channels in adult DRG neurons. Mol. Brain Res. 67, 267–282. 10.1016/s0169-328x(99)00070-4 10216225

[B13] Garrison S. R. Dietrich A. Stucky C. L. (2012). TRPC1 contributes to light-touch sensation and mechanical responses in low-threshold cutaneous sensory neurons. J. Neurophysiol. 107, 913–922. 10.1152/jn.00658.2011 22072513 PMC3289471

[B14] Ghitani N. Barik A. Szczot M. Thompson J. H. Li C. Le Pichon C. E.. (2017). Specialized mechanosensory nociceptors mediating rapid responses to hair pull. Neuron 95, 944.e4–954.e4. 10.1016/j.neuron.2017.07.024 28817806 PMC5599122

[B15] Häring M. Zeisel A. Hochgerner H. Rinwa P. Jakobsson J. E. T. Lönnerberg P.. (2018). Neuronal atlas of the dorsal horn defines its architecture and links sensory input to transcriptional cell types. Nat. Neurosci. 21, 869–880. 10.1038/s41593-018-0141-1 29686262

[B16] Honoré E. (2007). The neuronal background K_2P_ channels: focus on TREK1. Nat. Rev. Neurosci. 8, 251–261. 10.1038/nrn2117 17375039

[B17] Hwang H. Y. Zhang E. Park S. Chung W. Lee S. Kim D. W.. (2015). TWIK-related spinal cord K^+^ channel expression is increased in the spinal dorsal horn after spinal nerve ligation. Yonsei Med. J. 56, 1307–1315. 10.3349/ymj.2015.56.5.1307 26256973 PMC4541660

[B18] Kollert S. Dombert B. Döring F. Wischmeyer E. (2015). Activation of TRESK channels by the inflammatory mediator lysophosphatidic acid balances nociceptive signalling. Sci. Rep. 5:12548. 10.1038/srep12548 26224542 PMC4519772

[B19] Koltzenburg M. Stucky C. L. Lewin G. R. (1997). Receptive properties of mouse sensory neurons innervating hairy skin. J. Neurophysiol. 78, 1841–1850. 10.1152/jn.1997.78.4.1841 9325353

[B20] Lafrenière R. G. Cader M. Z. Poulin J. F. Andres-Enguix I. Simoneau M. Gupta N.. (2010). A dominant-negative mutation in the TRESK potassium channel is linked to familial migraine with aura. Nat. Med. 16, 1157–1160. 10.1038/nm.2216 20871611

[B21] Lawson S. N. Crepps B. Perl E. R. (2002). Calcitonin gene-related peptide immunoreactivity and afferent receptive properties of dorsal root ganglion neurone in guinea-pigs. J. Physiol. 540, 989–1002. 10.1113/jphysiol.2001.013086 11986384 PMC2290282

[B22] Lechner S. G. Lewin G. R. (2013). Hairy sensation. Physiology 28, 142–150. 10.1152/physiol.00059.2012 23636260

[B23] Li L. Rutlin M. Abraira V. E. Cassidy C. Kus L. Gong S.. (2011). The functional organization of cutaneous low-threshold mechanosensory neurons. Cell 147, 1615–1627. 10.1016/j.cell.2011.11.027 22196735 PMC3262167

[B24] Mathie A. Veale E. L. (2015). Two-pore domain potassium channels: potential therapeutic targets for the treatment of pain. Pflugers Arch. 467, 931–943. 10.1007/s00424-014-1655-3 25420526

[B25] McCoy E. S. Taylor-Blake B. Street S. E. Pribisko A. L. Zheng J. Zylka M. J. (2013). Peptidergic CGRPα primary sensory neurons encode heat and itch and tonically suppress sensitivity to cold. Neuron 78, 138–151. 10.1016/j.neuron.2013.01.030 23523592 PMC3628403

[B26] Nagy J. I. Hunt S. P. (1982). Fluoride-resistant acid phosphatase-containing neurones in dorsal root ganglia are separate from those containing substance P or somatostatin. Neuroscience 7, 89–97. 10.1016/0306-4522(82)90155-5 6176904

[B27] Peng C. Li L. Zhang M. D. Bengtsson Gonzales C. Parisien M. Belfer I.. (2017). MIR-183 cluster scales mechanical pain sensitivity by regulating basal and neuropathic pain genes. Science 356, 1168–1172. 10.1126/science.aam7671 28572455

[B28] Plant L. D. (2012). A role for K2P channels in the operation of somatosensory nociceptors. Front. Mol. Neurosci. 5:21. 10.3389/fnmol.2012.00021 22403526 PMC3293133

[B29] Ranade S. S. Woo S. H. Dubin A. E. Moshourab R. A. Wetzel C. Petrus M.. (2014). Piezo2 is the major transducer of mechanical forces for touch sensation in mice. Nature 516, 121–125. 10.1038/nature13980 25471886 PMC4380172

[B30] Royal P. Andres-Bilbe A. Ávalos Prado P. Verkest C. Wdziekonski B. Schaub S.. (2019). Migraine-associated TRESK mutations increase neuronal excitability through alternative translation initiation and inhibition of TREK. Neuron 101, 232.e6–245.e6. 10.1016/j.neuron.2018.11.039 30573346

[B31] Sano Y. Inamura K. Miyake A. Mochizuki S. Kitada C. Yokoi H.. (2003). A novel two-pore domain K^+^ channel, TRESK, is localized in the spinal cord. J. Biol. Chem. 278, 27406–27412. 10.1074/jbc.m206810200 12754259

[B32] Sehgal S. A. Hassan M. Rashid S. (2014). Pharmacoinformatics elucidation of potential drug targets against migraine to target ion channel protein KCNK18. Drug Des. Devel. Ther. 8, 571–581. 10.2147/dddt.s63096 24899801 PMC4038526

[B33] Silverman J. D. Kruger L. (1990). Selective neuronal glycoconjugate expression in sensory and autonomic ganglia: relation of lectin reactivity to peptide and enzyme markers. J. Neurocytol. 19, 789–801. 10.1007/bf01188046 2077115

[B34] Steinberg E. A. Wafford K. A. Brickley S. G. Franks N. P. Wisden W. (2015). The role of K_2_P channels in anaesthesia and sleep. Pflugers Arch. 467, 907–916. 10.1007/s00424-014-1654-4 25482669 PMC4428837

[B35] Stucky C. L. Lewin G. R. (1999). Isolectin B_4_-positive and -negative nociceptors are functionally distinct. J. Neurosci. 19, 6497–6505. 10.1523/JNEUROSCI.19-15-06497.1999 10414978 PMC6782829

[B36] Tulleuda A. Cokic B. Callejo G. Saiani B. Serra J. Gasull X. (2011). TRESK channel contribution to nociceptive sensory neurons excitability: modulation by nerve injury. Mol. Pain 7:30. 10.1186/1744-8069-7-30 21527011 PMC3095542

[B37] Usoskin D. Furlan A. Islam S. Abdo H. Lönnerberg P. Lou D.. (2015). Unbiased classification of sensory neuron types by large-scale single-cell RNA sequencing. Nat. Neurosci. 18, 145–153. 10.1038/nn.3881 25420068

[B38] Valenzuela D. M. Murphy A. J. Frendewey D. Gale N. W. Economides A. N. Auerbach W.. (2003). High-throughput engineering of the mouse genome coupled with high-resolution expression analysis. Nat. Biotechnol. 21, 652–659. 10.1038/nbt822 12730667

[B39] Wang F. Flanagan J. Su N. Wang L. C. Bui S. Nielson A.. (2012). RNAscope: a novel in situ RNA analysis platform for formalin-fixed, paraffin-embedded tissues. J. Mol. Diagn. 14, 22–29. 10.1016/j.jmoldx.2011.08.002 22166544 PMC3338343

[B40] Weir G. A. Cader M. Z. (2011). New directions in migraine. BMC Med. 9:116. 10.1186/1741-7015-9-116 22027350 PMC3217871

[B41] Weir G. A. Middleton S. J. Clark A. J. Daniel T. Khovanov N. McMahon S. B.. (2017). Using an engineered glutamate-gated chloride channel to silence sensory neurons and treat neuropathic pain at the source. Brain 140, 2570–2585. 10.1093/brain/awx201 28969375 PMC5841150

[B42] Wright P. D. Weir G. Cartland J. Tickle D. Kettleborough C. Cader M. Z.. (2013). Cloxyquin (5-chloroquinolin-8-ol) is an activator of the two-pore domain potassium channel TRESK. Biochem. Biophys. Res. Commun. 441, 463–468. 10.1016/j.bbrc.2013.10.090 24383077

[B43] Yoo S. Liu J. Sabbadini M. Au P. Xie G. X. Yost C. S. (2009). Regional expression of the anesthetic-activated potassium channel TRESK in the rat nervous system. Neurosci. Lett. 465, 79–84. 10.1016/j.neulet.2009.08.062 19716403 PMC2778220

[B44] Zeisel A. Hochgerner H. Lönnerberg P. Johnsson A. Memic F. van der Zwan J.. (2018). Molecular architecture of the mouse nervous system. Cell 174, 999.e22–1014.e22. 10.1016/j.cell.2018.06.021 30096314 PMC6086934

[B45] Zhou J. Yang C. X. Zhong J. Y. Wang H. B. (2013). Intrathecal TRESK gene recombinant adenovirus attenuates spared nerve injury-induced neuropathic pain in rats. Neuroreport 24, 131–136. 10.1097/wnr.0b013e32835d8431 23370493

